# Clinical Features and Outcomes of Adrenal Cavernous Hemangioma: A Study of 8 Cases from a Single Center

**DOI:** 10.1155/2021/5549925

**Published:** 2021-04-28

**Authors:** Henghai Huang, Xiaoyun Wu, Xiaocao Lin, Delin Li, Jingjing Zeng

**Affiliations:** ^1^Department of Urology, Wuzhou GongRen Hospital, Wuzhou, GuangXi, China; ^2^Department of Nursing, GuangXi Medical College, Nanning, GuangXi, China; ^3^Department of Pathology, The First Affliated Hospital of GuangXi Medical University, Nanning, GuangXi, China

## Abstract

**Background:**

Cavernous hemangioma is a rare benign tumor that develops from the adrenal glands. In this study, we present our experience with patients with adrenal cavernous hemangiomas (ACH) in a Chinese population.

**Methods:**

Demographic, diagnostic, surgical, and pathological findings in patients at a single institution who were adrenalectomized as a result of ACH were retrospectively reviewed.

**Results:**

Among 601 patients who underwent adrenalectomy, 8 (1.33%; 5 men, 3 women) cases were diagnosed with ACH between January 1, 1998, and December 31, 2018, in a single institution. The mean age was 53.25 ± 11.9 years (range, 35–67 years). Four (50%) were asymptomatic, and three (37.5%) complained of abdominal or flank discomfort. Preoperative computed tomography (CT) revealed ACH in 3 (37.5%) cases. Well-defined borders and heterogeneous enhancement with characteristic progressive partial filling-in were characteristic CT features of ACH (tumor size>3 cm). The mean tumor size was 5.16 ± 3.4 cm (range, 1.5–11 cm). No recurrence occurred during a median follow-up period of 38.37 months (range, 8–60 months).

**Conclusions:**

ACH was asymptomatic in most cases, and diagnosis could be challenging. Adrenalectomy is a safe treatment modality for ACH, and it ensures favorable outcomes.

## 1. Introduction

Cavernous hemangioma is a benign neoplasm originating from cavernous anomalous vessels composed of many thin-walled vessels. This neoplasm mostly occurs in the liver and kidney [1–3].

Adrenal cavernous hemangioma (ACH) is rare in clinical practice [4]. Most previous studies have addressed heterogeneous groups of hemangiomas and did not focus on ACH. There are approximately 90 cases currently reported in the literature [4–7]; however, most of them are case reports and series [8–10]. Furthermore, the small numbers of cases included in these studies have limited a comprehensive understanding of ACH. The detailed incidence of ACH in patients undergoing adrenalectomy remains unknown. Symptoms related to hormonal abnormalities, major surgery-related complications, mortalities, and recurrences have rarely been reported. ACH is usually benign and slow growing. It is usually asymptomatic, but some patients can present with either abdominal or flank pain. It has a good prognosis after surgical excision.

To further understand ACH and summarize the clinical features, we have shared our experiences regarding the clinical features, diagnostic imaging, and operative treatments of 8 ACH patients in this study.

## 2. Materials and Methods

Between January 1, 1998, and December 31, 2018, 601 patients with adrenal neoplasms were treated at the Wuzhou GongRen Hospital. Of these, 8 cases (1.33%, 8/601) were pathologically diagnosed as having ACH. The clinicopathological features, diagnosis, operation, and follow-up data of these patients were retrospectively analyzed. This study was approved by the Ethics Committee of Wuzhou GongRen Hospital.

The demographic parameters, chief complaints, and imaging features were analyzed. All of the patients were assessed for adrenal function, including (1) serum potassium; (2) plasma adrenocorticotropic hormone and cortisol (8:00 am, 4:00 pm, and 12:00 am); (3) 24-hour urinary free cortisol; (4) plasma renin and aldosterone (standing and lying positions); (5) serum epinephrine, norepinephrine, and dopamine; (6) 24-hour 3-methyl-4-hydroxymandelic acid (VMA); and (7) serum testosterone and estrogen.

All of the patients were evaluated by ultrasound and enhanced CT. The precontrast and postcontrast Hounsfield units (HU), maximum diameter, characteristics, and preoperative diagnosis were analyzed.

Surgical resections were obtained from patients with the following characteristics: tumor size more than 3 cm, smaller tumors enlarging by at least 1 cm per year, and tumors with imaging changes suggesting hemorrhage or calcification. Surgical treatment was performed by 2 surgeons with comparable seniority. Specific procedures included open and laparoscopic surgery depending on the preoperative diagnosis, tumor size, patient willingness, and surgeon preference. The operation time (OT), estimated blood loss (EBL), postoperative days, and complications were evaluated.

The surgical specimens were confirmed by pathological examination. The surgical margins and pathological diagnosis were evaluated by two pathologists specializing in urological diseases. All of the patients were followed up regularly every 3 months and by computed tomography (CT) every 6–12 months after surgery.

## 3. Results

Table 1 summarizes the demographic characteristics and clinical manifestations. All of the patients underwent preoperative adrenal endocrine function examination. Preoperative hormone and serum potassium levels were normal. The mean age was 53.25 ± 11.9 years (range, 35–67 years), including 6 patients with tumors on the left and 2 on the right. Four patients were incidentally detected; 3 presented with abdominal or flank pain, and 1 patient had hypertension.

All of the adrenal masses were unilateral. The mean preoperative size was 4.85 ± 3.47 cm (range 1.2–10.2 cm). The tumor size in 5 patients was more than 3 cm, and in 3 patients, it was less than 3 cm. Ultrasonography and CT were performed in 8 patients. Ultrasonography showed no specificity. The masses showed solid or mixed echoes. Blood flow signals could be seen in the tumors.

**Figure 1:**
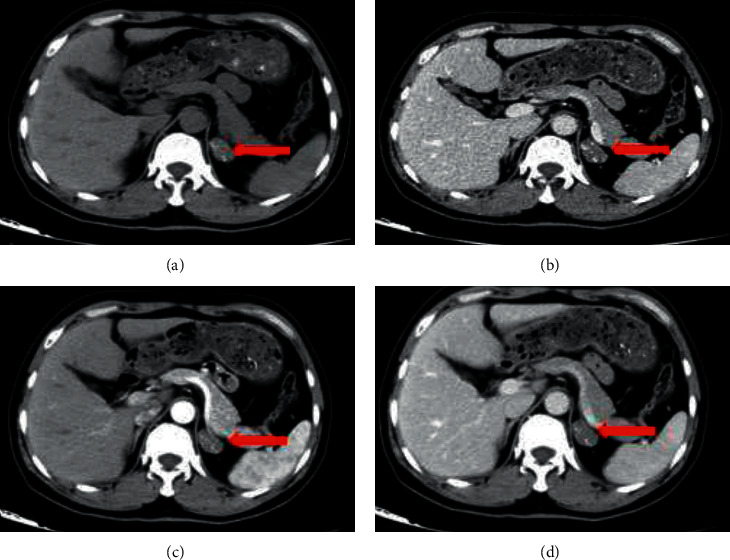
Left adrenal cavernous hemangioma with a tumor size of 2 cm. CT imaging showing an oval, well-defined, obvious calcified mass with mild enhancement (red arrow) in the left adrenal region. (a) CT unenhanced. (b) Arterial phase of CT. (c) Venous phase of CT. (d) Delayed phase of CT.

Calcification was found in 4 patients (50%, 4/8) who underwent enhanced CT scanning. Only 3 patients (37.5%, 3/8) were diagnosed with ACH before surgery. When the tumor size was smaller than 3 cm, a CT scan showed low-density or isodense tumors, and spot and nodular calcifications could be seen in the tumors, mildly enhanced on scanning (Figure 1).

**Figure 2:**
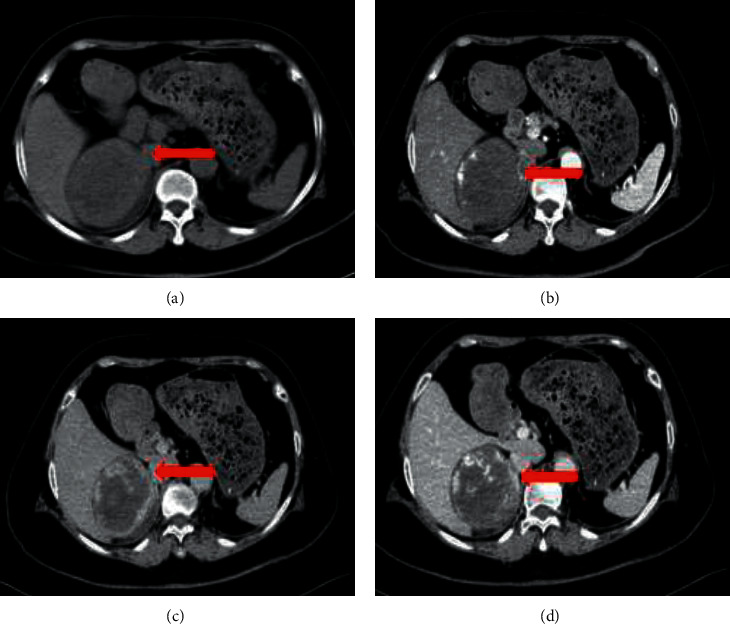
Right adrenal cavernous hemangioma with a tumor size of 8 cm. (a) The unenhanced CT image. (b) Enhanced multiphase CT images revealing early peripheral nodular enhancement in the arterial phase (red arrow). (c), (d) Progressive partial filling-in (red arrow) in the venous phase and delayed phase.

**Figure 3:**
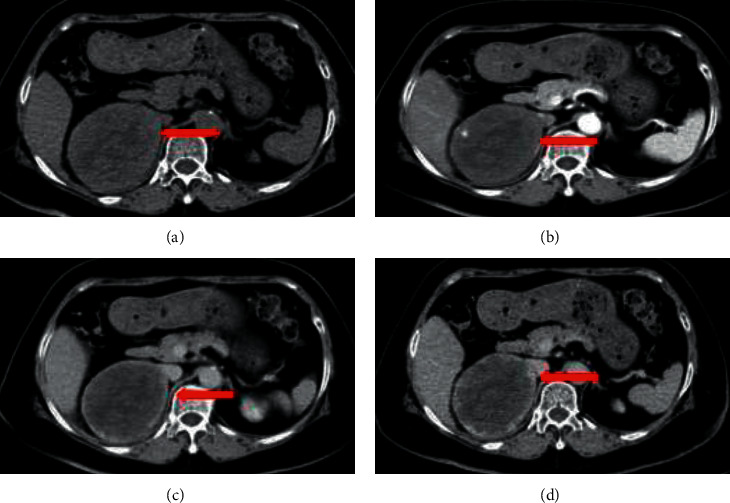
Right adrenal cavernous hemangioma with a tumor size of 10 cm. CT showing mild heterogeneous enhancement of the tumor during the arterial phase and progressive enhancement in the venous phase and the delayed phase (red arrow). (a) Enhanced CT. (b) Arterial phase of CT. (c) Venous phase of CT. (d) Delayed phase of CT.

When the tumor size was larger than 3 cm, CT scans revealed tumors with different enhancement patterns. Typical manifestations included concentric enhancement. Unenhanced CT imaging showed oval, well-demarcated, heterogeneous masses (Figure 2(a)). Peripheral enhancement in the arterial phase, enhancement degree of the venous phase, and the delayed phase extended from peripheral to central (Figures 2(b) and 2(d)). Other patterns included mild heterogeneous enhancement of the tumor during the arterial phase and progressive enhancement in the venous and delayed phases (Figure 3). CT scans showed no evidence of surrounding tissue infiltration or regional lymph node enlargement.

All 8 patients underwent surgical resection. Three (8 cm, 8 cm, and 11 cm) were diagnosed with static adrenal pheochromocytoma before surgery. *α*-receptor blockers were given 7–14 days before surgery. Retroperitoneal laparoscopic adrenalectomy was performed in 5 cases (tumor sizes 3.5 cm, 3.2 cm, 1.5 cm, 4.1 cm, and 2 cm). The mean OT was 51 min (range 30–70 min). The mean EBL was 110 ml (range 80–150 ml). The mean hospital stay was 4.2 days (range, 3–5 days).

Three cases (tumor sizes 8 cm, 10 cm, and 11 cm) were performed by open adrenalectomy. The mean OT was 110 mins (range, 100–120 min). The mean EBL was 283 ml (range 200–350 ml). The mean hospital stay was 6.3 days (range 6-7 days).

There were no deaths, minor morbidities, or complications during the perioperative period. During a mean follow-up of 38.37 months (range, 8–60 months), no patients showed recurrence or metastasis on serial imaging evaluations.

**Figure 4:**
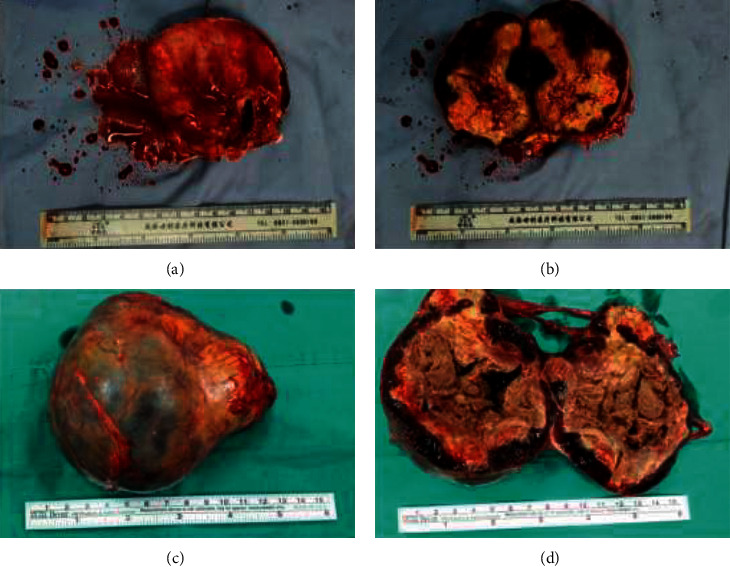
The specimen of ACH. (a-b) Gross appearance revealing a completely encapsulated and moderately firm round mass measuring 8 × 6 × 6 cm, with a cross section of reddish-brown and ash-gray organized hematoma. (c-d) Pathological examination revealed a resected oval mass measuring 11 × 8 × 6 cm, which was spongy and yellowish with a central area, whitish peripheral capsule, and hard consistency.

**Figure 5:**
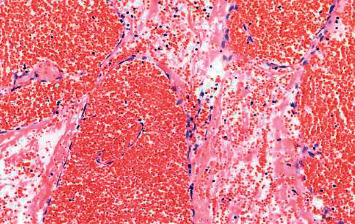
Microscopically, a typical adrenal cavernous hemangioma, including several dilated interconnecting vascular channels with thrombosis (hematoxylin and eosin staining ×200, original magnification).

The mean tumor size was 5.16 ± 3.4 cm (range, 1.5–11 cm). Gross specimens showed that the masses appeared encapsulated with cystic-solid content. The textures were medium. The cut surfaces were yellowish brown, brown, or dark red, with dilated sinuses or bleeding (Figure 4). Microscopically, adrenal tissues were replaced by proliferative and dilated vessels, with irregular vessels, thin walls, monolayer epithelial cells, and fibrous cystic wall formation (Figure 5). All 8 patients were diagnosed with ACH by pathology.

## 4. Discussion

Approximately 90 cases have been reported in the literature since ACH was first reported in 1955. The ratio of male to female patients is approximately 2:3; the mean age is 49.2 years [4]. The incidence is mostly unilateral and rarely bilateral [11]. In this study, the mean age was 53.25 years, consistent with the literature review. Furthermore, the majority of cases were likely isolated; therefore, the detailed incidence of ACH in patients undergoing adrenalectomy remains unknown. In this study, the incidence of ACH among these patients was 1.33% (8/601) in our institution.

Most of the patients had no clinical symptoms. They only presented for diagnosis of adrenal lesions by imaging examinations or because of other diseases. Some patients complained of corresponding symptoms, including flank or abdominal pain and abdominal distension in adjacent organs due to tumor volume enlargement. It has also been reported that ACH can present with rupture hemorrhage and shock symptoms, including decreased blood pressure, which can lead to death in severe cases. The literature mentions that the average tumor size was 4.8 cm [4] and the maximum tumor size was 42 cm [10]. The mean tumor size in our series was 5.26 cm, which was larger than that reported in the literature, possibly related to the late presentations of the patients.

It is difficult to diagnose ACH before surgery. Imaging findings have certain significance for ACH diagnosis [12, 13]. When the tumor size is more than 3 cm, a CT scan can reveal tumors showing various enhancement patterns. A typical manifestation was concentric enhancement [4]. Tumors smaller than 3 cm in diameter do not have this imaging feature. It was only indicated that the tumor was mildly enhanced. On imaging, ACH must be differentiated from adrenal schwannoglioma, adrenocortical carcinoma, and hepatic hemangioma [14, 15].

The final diagnosis of ACH depends on histopathology. Microscopic examination of ACH revealed dense and dilated blood vessels that were arranged in a disorderly fashion. Some vasodilatation and congestion were sponge-like, and the walls were thin. The tumors are usually composed of single-layer endothelial cells, forming sinusoids of various shapes and sizes. The wall of the tube could have a ground-glass appearance. Some tubes showed combined calcification and fiber capsule wall formation.

Most cases of ACH have no endocrine function. To date, only 3 cases have been reported to have endocrine function [16, 17]. It is recommended to perform routine adrenal endocrine examinations before surgery to determine the nature of adrenal incidental tumors and to establish the presence or absence of endocrine function to choose the appropriate treatment.

The treatment of ACH depends on the size of the tumor. Currently, the literature holds that when the tumor diameter is >3.5 cm, the risk of spontaneous bleeding increases; therefore, active surgical treatment is recommended. Patients with adrenal hemangiomas <3.5 cm can be closely observed; if there is tumor enlargement, surgery can be considered [5, 9, 18].

Laparoscopic adrenalectomy is recommended when the tumor size is smaller than 6 cm [19, 20]. Because of improvements in laparoscopic instruments and technical expertise, there have been reports of large tumors (more than 10 cm) that have been successfully removed by the laparoscopic approach [21]. Therefore, the size of the tumor is no longer a limitation of laparoscopic surgery. Specific operation patterns were performed according to the situation of the patients and the surgeon's preference and technical expertise. In this study, retroperitoneal laparoscopic adrenalectomy was performed in 5 patients with tumor sizes smaller than 6 cm, whereas open surgery was performed in 3 patients with tumor sizes larger than 6 cm.

ACH has a good prognosis. The tumors were completely resected with no deaths or complications despite the large sizes of the masses [5, 22, 23].

However, based on our limited data, the biological characteristics of ACH are still not entirely clear. Therefore, a regular follow-up schedule is recommended for patients after surgery, including CT scanning and routine endocrine testing.

In conclusion, ACH is a rare benign tumor occurring mostly in middle-aged adults. The preoperative diagnosis of ACH remains difficult. Clinicians should always meticulously suspect ACH when enhanced CT shows an adrenal tumor with concentric enhancement. The final diagnosis depends on histopathology. After complete resection, the prognosis of ACH patients is excellent.

## Figures and Tables

**Table 1:** Patients' demographic and clinical characteristics.

Characteristics	Value
*Mean age in years at diagnosis (range)*	53.25 (35–67)

*Gender*	
Female (%)	3 (37.5%)
Male (%)	5 (62.5%)

*Clinical presentations*	
Incidental (%)	4 (50%)
Abdominal/back discomfort (%)	3 (37.5%)
High blood pressure (%)	1 (12.5%)

*Tumor site*	
Left	6 (75%)
Right	2 (25%)

Mean preoperative tumor size in cm (range)	4.85 ± 3.47 cm
Mean tumor size in cm on pathology (range)	5.16 ± 3.4 cm

## Data Availability

The datasets used and analyzed during the current study are available from the corresponding author on reasonable request.
